# Supplementary material for: A Global Screen for Assembly State Changes of the Mitotic Proteome by SEC-SWATH-MS
Source: Cell Syst. 2020 Feb 26;10(2):133–155.e6. doi: 10.1016/j.cels.2020.01.001 (PMC7042714; doi:10.1016/j.cels.2020.01.001)

Q9UPR0 | PLCL2_HUMAN | PLCL2 KIAA1092 PLCE2
Monomer MW [kDa]: 125.866 Monomer expected elution fraction: 38

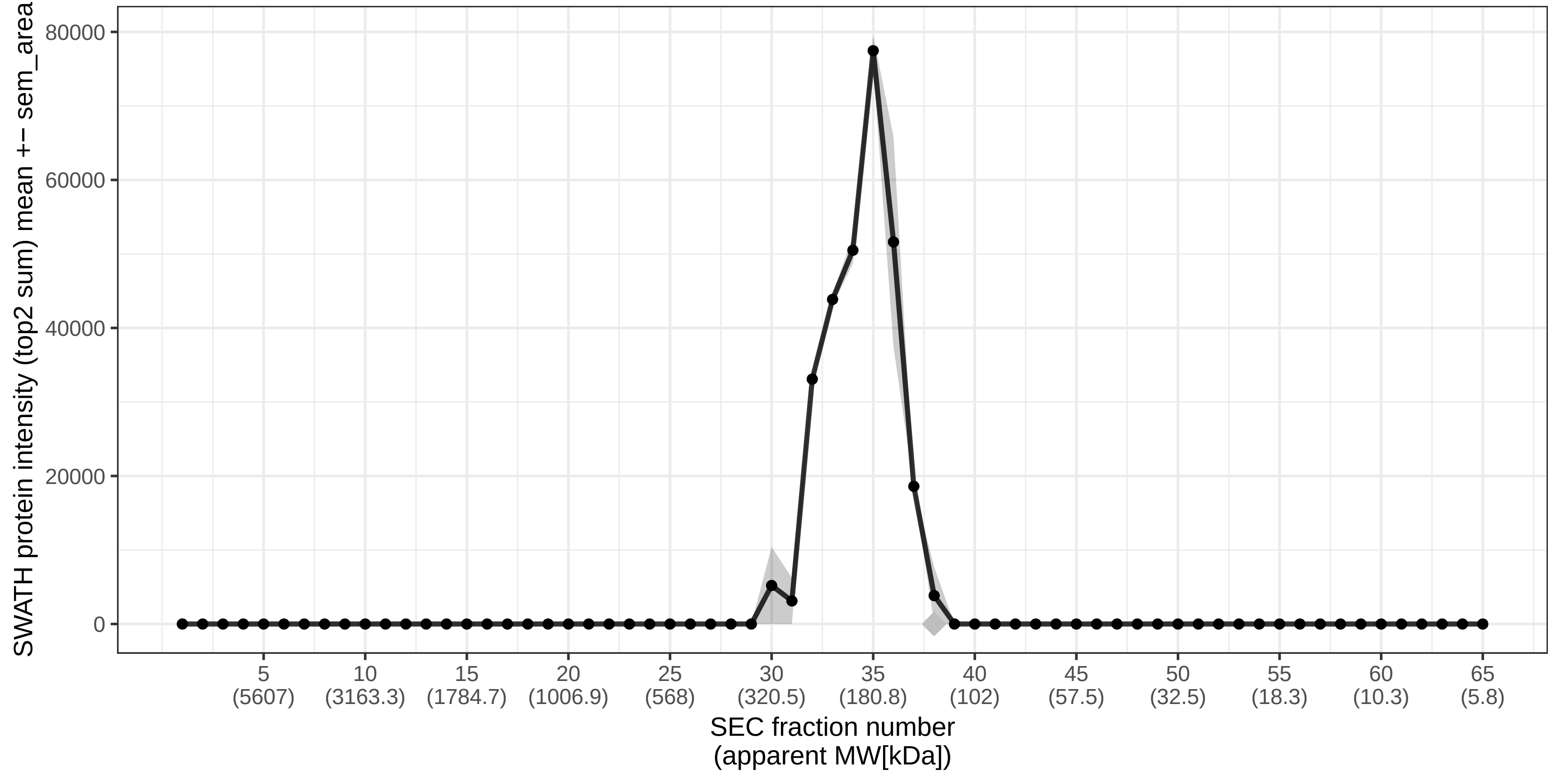

Supplement: Data S1. SEC-SWATH-MS Protein Chromatograms, Related to Figure 1 [file mmc6.zip › SECchrom_Q9UPR0_PLCL2_HUMAN_PLCL2_KIAA1092_PLCE2.pdf]
